# Society Meeting

**Published:** 1909-08

**Authors:** 


### SOCIETY MEETING.

Buffalo Academy of Medicine Officers for 1909-10: president, Earl P. Lothrop; secretary, Harry R. Trick; treasurer, Lawrence Hendee; trustee, for three years, Grover Wende.

Section Officers. Medicine: chairman, Thomas J. Walsh; secretary, Henry P. Frost.

Surgery: chairman, Thew Wright; secretary, James A. McLeod.

Obstetrics and Gynecology. chairman, Burt C. Johnson; secretary, J. F. Whitwell.

Pathology: chairman, Fred J. Parmenter; secretary, Norman K. McLeod.

